# Cosolvent and Dynamic Effects in Binding Pocket Search by Docking Simulations

**DOI:** 10.1021/acs.jcim.1c00924

**Published:** 2021-11-03

**Authors:** P. Bernát Szabó, Francesc Sabanés Zariquiey, Juan J. Nogueira

**Affiliations:** † Department of Chemistry, KU Leuven, Celestijnenlaan 200F, 3001 Leuven, Belgium; ‡ Department of Chemistry, Universidad Autónoma de Madrid, Calle Francisco Tomás y Valiente, 7, 28049 Madrid, Spain; § IADCHEM, Institute for Advanced Research in Chemistry, Universidad Autónoma de Madrid, Calle Francisco Tomás y Valiente, 7, 28049 Madrid, Spain

## Abstract

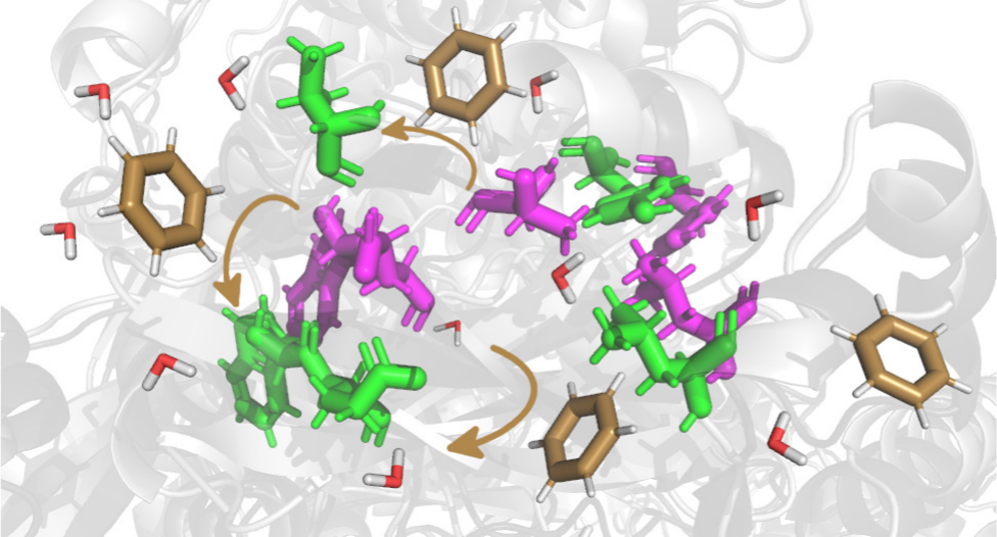

The lack of conformational sampling in virtual screening projects can lead to inefficient results because many of the potential drugs may not be able to bind to the target protein during the static docking simulations. Here, we performed ensemble docking for around 2000 United States Food and Drug Administration (FDA)-approved drugs with the RNA-dependent RNA polymerase (RdRp) protein of severe acute respiratory syndrome coronavirus 2 (SARS-CoV-2) as a target. The representative protein structures were generated by clustering classical molecular dynamics trajectories, which were evolved using three solvent scenarios, namely, pure water, benzene/water and phenol/water mixtures. The introduction of dynamic effects in the theoretical model showed improvement in docking results in terms of the number of strong binders and binding sites in the protein. Some of the discovered pockets were found only for the cosolvent simulations, where the nonpolar probes induced local conformational changes in the protein that lead to the opening of transient pockets. In addition, the selection of the ligands based on a combination of the binding free energy and binding free energy gap between the best two poses for each ligand provided more suitable binders than the selection of ligands based solely on one of the criteria. The application of cosolvent molecular dynamics to enhance the sampling of the configurational space is expected to improve the efficacy of virtual screening campaigns of future drug discovery projects.

## Introduction

Proteins are ubiquitous building blocks playing a critical role in the reproduction, metabolism, and regulation of living organisms and viruses. Understanding and manipulating the way proteins interact with their surrounding is, therefore, of utmost interest from both biological and medical points of view. Currently, the most important method to manipulate the function of proteins is through the administration of drugs. For this reason, there exists a growing interest in identifying new binders for a wide variety of proteins in the hopes of treating a number of different diseases.^1−7^ In fact, 78% of the biological drugs approved by the United States Food and Drug Administration (FDA) have clear protein molecular targets.^8^ Therefore, it is not surprising that scientists turned to them once again when faced with the new and immediate challenges of the coronavirus disease 2019 (COVID-19).

The COVID-19 pandemic caused by the severe acute respiratory syndrome coronavirus 2 (SARS-CoV-2) continues to claim thousands of lives every day more than a year after its outbreak.^9^ However, the knowledge and the developed tools to fight against it are vastly more potent than they were a year before.^10^ Antiviral drugs targeting the proteins vital to the reproduction of SARS-CoV-2 have been the most important tools,^11^ aside from vaccines that are being used as preventative measures. For example, Remdesivir, one of the most widely used antiviral drugs against SARS-CoV-2 around the world,^12^ targets the RNA-dependent RNA polymerase (RdRp) protein of the virus.^13^ Furthermore, given the urgency of developing an effective treatment, most attempts to find new inhibitor substances were in fact drug repurposing studies, targeting the virus’s RdRp^14−18^ or other important proteins.^19−22^ The RdRp protein is an especially promising drug target as it is responsible for the replication of the viral RNA inside the host cell,^23^ and it is highly similar to the RdRp of SARS-CoV,^24^ which already has a number of verified inhibitors.^25^ In addition, its high-quality three-dimensional (3D) structure has been available from as early as April 2020.^26^ In large part, due to the urgent nature of the COVID-19 pandemic, most of the above-cited research projects relied heavily, or even exclusively, on computational techniques for the discovery of the potential inhibitors, due to the cost and time efficiency of such methods.

High-throughput screening enables routine evaluation of thousands of substances in a week.^27^ This tremendous efficacy is often supported by the development and application of innovative computational methods, which became more useful since the advent of structure-based drug design, where potential drugs are created or found based on the 3D structure of the protein target.^28,29^ Although such target structures were initially only obtainable through costly and cumbersome experimental methods, such as X-ray crystallography^30^ or nuclear magnetic resonance (NMR) spectroscopy,^31^ they are nowadays much more readily available due to the gradual improvement of existing methods and the development of new experimental methods, such as cryo-electron microscopy.^32^ The information obtained by experimental techniques can be complemented and extended by the application of well-established computational techniques, such as homology modeling^33^ and ensemble docking,^34^ or by more advanced and recent methods based on artificial intelligence.^35,36^ By employing one (or a combination) of the above techniques, high-quality structures are available for a larger number of protein targets than ever before.

The current challenge to computational chemists is therefore how to best utilize the available structural information. The computational methods developed for structure-based drug design fall into two main categories: *de novo* design methods construct new, tailored ligands, while docking methods select ligands complementary to the target from the existing compound space.^37^ Although different methodologies have been widely employed to investigate the binding of ligands to proteins, such as Monte Carlo techniques^38,39^ and Gaussian accelerated molecular dynamics (MD),^40,41^ the vast majority of structure-based virtual screening (VS) investigations rely on docking calculations due to their computational efficiency.^7,42^ This procedure can be thought of as a computational complement to high-throughput screening, where a large number of compounds are docked to the target protein structure *in silico*. Traditionally, VS campaigns have been carried out utilizing a single, experimentally determined protein structure, often in the crystallized form.^37,43^ However, the deficiencies of using only a single crystallized protein structure have been recently recognized.^37,43−46^ First, the structure of the crystallized protein often differs significantly from the conformations that the protein adopts *in vivo*. Second, even if the crystal structure is representative of the conformation most often visited in solution, a single structure cannot account for the dynamics of protein motion.

Different theoretical models that consider the importance of protein motion have been developed at the cost of computational efficiency, *e.g*., the induced-fit model of ligand docking,^47,48^ where the structure of the protein may change during ligand uptake, or the model of conformational selection,^49−51^ which views the target protein as a dynamic object even in the absence of ligands. The need to take protein flexibility and motion into account became even clearer with the discovery of cryptic or hidden pocket structures.^52−54^ The characteristic property of these pockets is that they only appear in the presence of the appropriate ligand, while their existence is not obvious from the equilibrium structure of the protein. The exact mechanism of their formation is not yet clear, although some combination of induced-fit and conformational selection has been hypothesized.^53^ The discovery and theoretical description of such pockets are hindered by the fact that their opening often requires large-scale rearrangements of the protein structure, events that are traditionally hard to predict with standard computational techniques.^55^

With the importance of protein dynamics gaining wider recognition, new and more elaborate methods are appearing, which aim to account for this phenomenon. On the one hand, some of the modern computational docking programs, such as AutoDock Vina,^56^ can treat a selected number of protein residues as flexible at the cost of increased calculation times. This method is well suited to study a previously known, specific binding site of the protein. However, it cannot account for larger structural changes of the protein and is limited to a handful of flexible residues due to its computational requirements. On the other hand, the family of ensemble docking techniques utilizes traditional (rigid protein) docking calculations in combination with an ensemble of protein conformations to account for the flexibility of the target.^37,43^ The careful selection of the structures of the ensemble can enable the description of large-scale conformational changes and to the discovery of new cryptic pockets.^51,54,55^ The main challenge for these methods is the generation of the protein structure ensemble, which can be achieved experimentally using different crystallized structures,^37,57,58^ or computationally by, *e.g*., conformational space searches,^59^ neural networks,^60^ and MD.^43,61^

MD is an especially promising avenue, after all it has been designed for the very purpose of efficiently sampling the conformational space of proteins and other large molecules. However, one of the most important obstacles of MD calculations is the extremely slow convergence of the calculated trajectories,^43^ which precludes the population of rarely visited conformations. Even with highly specialized codes and computers, the longest timescales reachable are in the range of milliseconds.^62^ To be able to sample rare events, a number of modified MD techniques have been developed. The first group of these is the enhanced sampling methods, where some unphysical bias is introduced into the simulation to encourage the sampling of otherwise unlikely conformations. Some of the most popular enhanced sampling methods in the context of cryptic pocket discovery are umbrella sampling,^63^ steered MD,^64^ metadynamics,^65^ and replica-exchange MD,^66^ among others. A completely separate approach for the sampling of rarely visited conformations harboring cryptic pockets is that of the cosolvent methods. The main idea behind these frameworks is to replace the traditional water solvent in MD simulations with a mixture of water and some other cosolvent. The oftentimes hydrophobic or amphipathic cosolvent probes can then interact with the protein and occasionally induce conformational changes or stabilize some conformations where a cryptic pocket is open. Cosolvent methods have been successfully used to identify cryptic sites in a number of targets.^51,54,67−72^

The primary aim of the present work is to investigate the effect of protein dynamics in the results of a VS campaign. The ensemble of protein structures is obtained via MD simulations. Further sampling is achieved by cosolvent trajectories where water/benzene and water/phenol mixtures are employed. Recognizing the severity of the COVID-19 pandemic, the calculations are carried out on the RdRp protein of SARS-CoV-2 and a set of FDA-approved small-molecule drugs, in the hopes of contributing to the generation of knowledge necessary to develop effective treatments against this virus. We show that the introduction of protein dynamics in VS significantly improves the chances to find suitable binders and unveil potential new binding sites in the protein. This is especially relevant considering the significant amount of work published in the last year aimed to discover efficient antiviral agents against the SARS-CoV-2 virus based on static docking calculations that neglect dynamic effects.

## Computational Details

### Setup and Molecular Dynamics Simulations

The Wuhan-Hu-1 SARS-CoV-2 RdRp protein complex (PDB code 6M71, UNIPROT code P0DTD1) was chosen as the target of our investigations.^73^ In its active form, it is composed of three domains, namely, the nonstructural proteins (NSP) 7, 8, and 12 of SARS-CoV-2. Its active site is located in a deep groove at the boundary of the palm and thumb domains of its NSP12 unit,^14^ and is highly similar to that of the analogous protein of the SARS-CoV.^25^ Its simulation-ready structure was obtained from the website of D. E. Shaw Research,^74^ where extensive MD simulations have already been carried out for it. In ref (14), the authors note that two zinc ions could be necessary for the structural integrity of the protein. These ions were not found in the structures and trajectories downloaded from D. E. Shaw Research. However, the structure of the protein was maintained stable along the downloaded 10 μs trajectory in the absence of the ions. After numerous failed attempts at stabilizing these zinc ions in their bound positions with restraining potentials and gradual heating, and given the large stability shown by the protein, their inclusion was rejected in favor of the original D. E. Shaw structure. In addition, it is important to highlight that the main goal of the present work is to investigate the effect of the enhanced sampling obtained by cosolvent dynamics on docking simulations, and not to obtain all of the exact conformations that could be visited by the protein. Therefore, the use of dynamic trajectories without including the effect of the two zinc ions is sufficient for our purposes. Additionally, the crystal structure in its apo form determined with cryo-electron microscopy was also downloaded from the Protein Data Bank website (6M71)^73^ to carry out docking calculations, as explained below.

The MD calculations were carried out with the Amber 18 program package,^75^ according to the following protocol. The protein was described by the ff14SB force field,^76^ water by the TIP3P model,^77^ the cosolvents benzene and phenol by the general Amber force field for organic molecules,^78^ and the sodium ions by suitable Amber force field parameters.^79^ Three types of solvent boxes were prepared for the simulations: a simple water one and two with either benzene or phenol as cosolvent. The cosolvent boxes were built with the help of the packmol program.^80^ The concentration of the cosolvents was set to 10% v/v. In the case of benzene, severe clustering of the cosolvent molecules was observed during the MD simulations when the default force field parameters were used. To circumvent this issue, scripts included in the ParmEd distribution^81^ were utilized to introduce Lennard-Jones potentials for the C atoms of the benzene molecules with parameter values of γ = 0.00036 kcal/mol and *R*_min_ = 7.12719 Å (the default values used in ParmEd). After this modification was made, no clustering of the benzene molecules was observed during the simulations.

The protein structures were solvated using the tleap program of Amber 18^75^ in octahedral solvent boxes, where a distance of at least 12 Å was left between any atom of the protein and all sides of the solvent box. The charge of the system was neutralized with sodium ions. During the simulations, the particle–mesh Ewald method^82^ with a grid spacing of 1.0 Å was used to compute the electrostatic interactions, and a 12 Å cutoff for the nonbonded interactions was chosen. The solvated systems were first minimized for 1000 gradient descent steps followed by 1000 conjugate gradient steps. Next, heating of the systems to 300 K was performed during a 1 ns simulation with the Langevin thermostat (collision frequency of 1.0 ps^–1^) and with a 2.0 fs timestep in the NVT ensemble. Finally, production simulations were carried out at 300 K and 1 bar pressure using the Langevin thermostat (collision frequency of 1.0 ps^–1^) and Berendsen barostat (relaxation time of 2.0 ps) in the NPT ensemble, using a timestep of 2.0 fs. Specifically three replicas of 200 ns each were run for the production calculation for each solvent. The last two simulations for each solvent were started from a random equilibrated frame of the first simulation for that solvent, with the velocities of all particles randomized according to the Boltzmann distribution at 300 K. The production calculations were run with graphics processing unit (GPU) acceleration, using the CUDA version of the pmemd program of Amber 18.^83^

For the clustering of the MD trajectories the cpptraj program^84^ of Amber 18 was utilized. A density-based clustering algorithm (chosen with the dbscan keyword of cpptraj) was employed, with the parameters *k* (unitless) and ε set to 4 and 1.1 Å, respectively, as will be explained below and in the Supporting Information. For each type of solvent, the equilibrated part of the first production simulation and the two additional replicas were concatenated and the clustering was carried out separately for each solvent. Before clustering, the structures in every frame were aligned to each other by their α carbon atoms. The clustering was performed using the root-mean-square deviation (RMSD) values of the α carbon atoms as the distance metric between the conformations. A total of 19 cluster representatives were obtained with 13 structures coming from the trajectory with water as the solvent, while the benzene and phenol cosolvent trajectories yielded three cluster representatives each.

### Docking Calculations

**Figure 1:**
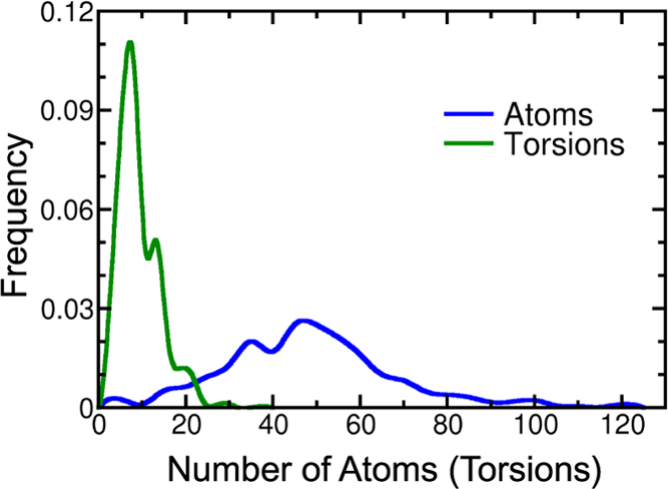
Probability distributions of the number of atoms and torsions present in the 1957 ligand structures employed in the docking simulations.

The set of FDA-approved drugs were downloaded from the ZINC database^85^ in the mol2 format. We did not consider all possible tautomeric and protomeric states of each drug but just the one that is available in the database. This set is a popular choice for drug repurposing studies^16−18^ and, with approximately 2000 contained ligands, it was feasible to perform docking calculations for all protein conformation/ligand pairs. From this set, 1957 ligand structures were converted to the pdb format with the openbabel program,^86^ while around 40 drugs failed to be converted into pdb format. These failed drugs were not considered in our simulations. Then, the pdb files were converted into pdbqt files by AutoDockTools 4,^87^ which are the files needed by AutoDock Vina^56^ containing partial charges and torsion angles. The size and flexibility of the selected ligands can be seen in Figure 1, where the probability distribution of the number of atoms and torsion angles are plotted. Specifically, the ligands present a large variety of sizes, most of them being between 20 and 80 atoms, while the number of flexible torsions ranges from 5 to 20.

The 19 cluster representative protein structures along with the apo crystal structure were aligned to each other by the RMSD distances between their α carbons. The protein and ligand structures were prepared for docking, relying on the scripts included in the AutoDockTools 4 distribution.^87^ The same docking region, generated by AutoDockTools 4, was used for all docking calculations, which encompassed the whole protein structure. To carry out the docking calculations, AutoDock Vina was run with the default command line options, except for the exhaustiveness and num_modes options. The first one was increased from the default value of 8 to 24, as suggested by the authors in the manual of AutoDock Vina^56^ for large docking regions. The time spent by the program on the search is heuristically chosen depending on the number of atoms and flexibility of the ligands. However, when the search space is large, as in this case, it is advisable to increase the number of runs by increasing the value of the exhaustiveness keyword to attain converged results. In the case of num_modes, the parameter was set to 20 to obtain the 20 best poses for each ligand. The parallel execution of the docking calculations was managed with in-house scripts. Attending to the results of the docking calculations the best ligands binding to each discovered pocket were selected based on two different criteria, namely, binding free energy and binding free energy gap between the two best poses of each ligand, which will be discussed below.

### Pocket Description

The binding sites of the protein, discovered through computational docking calculations, were analyzed with the mdpocket program,^88^ part of the fpocket distribution. To this end, the 19 cluster representative protein structures were aligned to each other by their α carbons and concatenated to create a mock trajectory readable by mdpocket. The regions of space which the discovered binding pockets occupy were selected manually, by inspecting the poses of the ligands binding to the pocket in question. Based on the suggestions of the mdpocket authors, large regions were selected for each pocket, encompassing all or almost all docked ligand poses. With the protein structures concatenated and the binding regions selected, mdpocket was run with the −S option, instructing the program to score pockets by their druggability. Among its results, mdpocket provides a number of pocket descriptors calculated for each frame of the supplied trajectory. From these descriptors, the pocket volume is utilized in the present study to discuss the stability of the pockets.

## Results and Discussion

### Equilibration of the Trajectories

**Figure 2:**
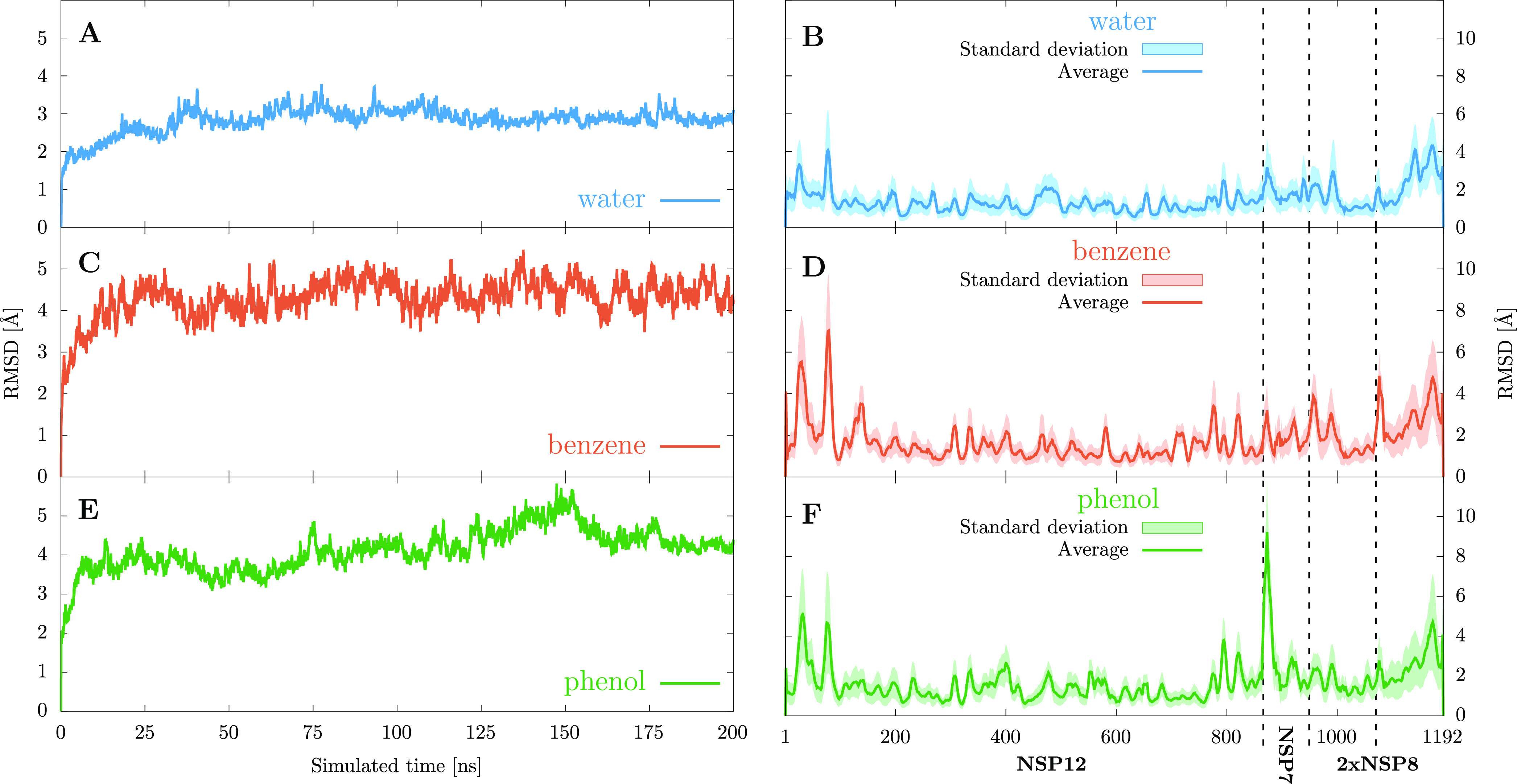
(A, C, E) Evolution of the RMSD distance of the protein from the starting conformation during the MD trajectories. The first replica for each solvent is plotted. (B, D, F) Average RMSD values for the protein residues in the three different solvent trajectories. The transparently colored region around the average values represents the standard deviation throughout the simulation. The first replica for each solvent is plotted. The location of the NSP domains of the protein is indicated by the dashed vertical lines.

In this section, first, the equilibration of the protein during the various (cosolvent) MD trajectories is examined by plotting the RMSD of the protein structure from its initial state throughout the simulated time. On top of the usual task of selecting the equilibrated part of each trajectory to be considered for further analysis, these plots are useful to detect potential differences in the equilibration process between the water solvent and the cosolvent trajectories. Figure 2A,C,E shows such plots obtained for the first replica of 200 ns of each solvent type. The protein structures are well equilibrated after 50 ns of simulation time in water and in benzene/water solvents. In the case of phenol/water, significant variations are still observed after 100 ns, a fact that could indicate that the cosolvent probes have stabilized some protein conformations that are not often visited with water as the solvent, and that are farther from the original protein conformation than those appearing frequently in water-based simulations. This is further investigated by plotting the average residue-wise RMSD values to monitor the mobility of the different regions of the protein. These data are shown in Figure 2B,D,F for the first production replicas of all solvents. The initial fraction of the trajectories where the RMSD suffers a strong increase, which correspond to 40, 20, and 10 ns for water, benzene/water, and phenol/water, was not considered. To make the plots more readable, the moving average of residue mobility calculated with a symmetric window with a width of nine residues is plotted. The numbering of the residues on this figure simply describes the order in which Amber 18 considers them, therefore these numbers cannot be straightforwardly compared with another numbering published in the literature.

At first glance, the three plotted curves of Figure 2B,D,F appear fairly similar: they showcase higher mobility of the terminal residues and lower average RMSD values for residues between 200 and 800. The section between residues 600 and 800 (the interface of the palm and thumb domains of the core NSP12 unit) is roughly where the active site of the protein is located. Since the conformation of the residues around the active site is crucial for the activity of the protein, it is not surprising that these regions are more stable in the absence of substrates than some other less critical areas. Upon more careful inspection, some differences between the three mobility plots can be identified. Most notable are the unusually high RMSD values around residues 870–880 observed in the trajectory with phenol as the cosolvent. These residues correspond to the terminal regions of the NSP12 and NSP7 units of the RdRp complex and, therefore, their higher mobility in itself is not surprising. The fact that the RMSD values are outstandingly high only in the phenol trajectory could indicate some occasional interaction of these residues with the phenol probes and could explain the higher RMSD observed in Figure 2E after 100 ns of simulation. A further difference between the three curves is the larger variance of the per residue RMSD values observed when the cosolvent MD trajectories are considered. First, this manifests itself in the slightly larger standard deviations shown for these trajectories, especially around residues 1100–1192, where larger standard deviation areas can be observed for the cosolvent trajectories with similar average mobility for all three trajectories. Second, the differences between the average RMSD values between neighboring residues are also in general larger for the two cosolvent trajectories. This results in much deeper valleys and higher, more pronounced spikes in Figure 2D,F than in Figure 2B. This latter observation can lead one to assume that the effects of the cosolvent probes are quite local in nature: they can significantly change the conformation of the handful of residues they are directly interacting with but leave the larger-scale structure of the protein more or less intact.

### Selection of Representative Protein Conformations

As mentioned above, the dbscan algorithm of cpptraj is used to perform the clustering of the trajectories. The clustering is carried out separately for the three solvents, with the three replicas of each of them concatenated and treated as a single trajectory, but excluding the first 40, 20, and 10 ns for water, benzene/water, and phenol/water, respectively, where the RMSD of the protein is not stabilized. To carry out a successful clustering of the trajectories, first, the *k* and ε parameters of the density-based clustering algorithm have to be tuned. On top of performing this tuning of the parameters, the effects of considering only the α carbon atoms for the RMSD calculations instead of all heavy atoms of the protein are also evaluated. The choice of the *k* and ε parameters and a comparison between the clustering when using only the α carbon atoms and when using all of the heavy atoms is deeply discussed in the Supporting Information. It was found that the values of ε = 1.1 Å and *k* = 4 are ideal choices and that considering only the α carbon atoms is enough to achieve a good clustering of the snapshots. The resulting clustering yielded 13, 3, and 3 representative protein conformations for the dynamics evolved in water, benzene/water, and phenol/water, respectively. These 19 conformations were utilized during the subsequent ensemble docking calculations.

Since the clustering of the trajectories obtained with different solvents is carried out independently from each other, it is possible that some cluster representatives coming from different trajectories are quite similar to each other. This redundancy would clearly not be optimal as it increases the computational requirements of the ensemble docking calculations without providing much additional information. It is worth investigating this redundancy as its presence would indicate that the cosolvent simulations do not provide protein conformations different than those from the simulation in pure water. To this end, another clustering is performed utilizing the parameters selected above, but with all trajectories considered at once. This clustering yields 18 cluster representative structures, which is only marginally less than the 19 ones obtained with the original clustering scheme. The fact that the clustering algorithm cannot merge many clusters coming from different solvent trajectories, *i.e*., returns a similar number of clusters as when the trajectories are considered individually, signals that the three trajectories with different solvents indeed visit markedly different conformations.

**Figure 3:**
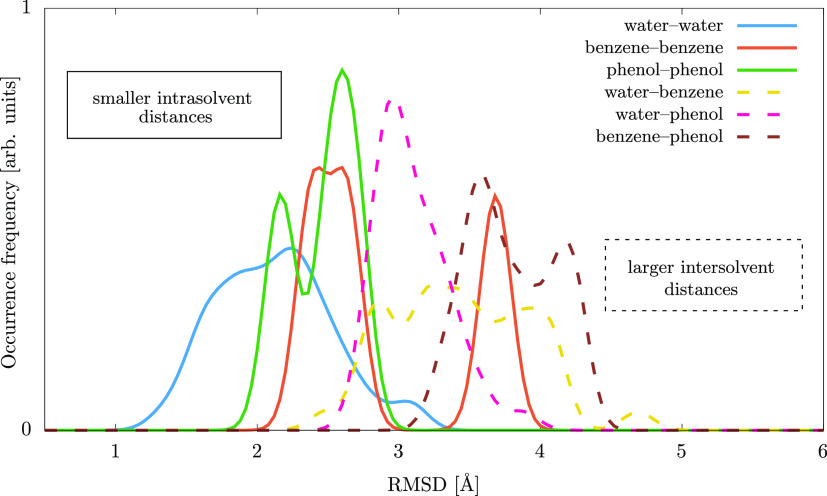
Distribution of the RMSD distances between the selected protein conformation cluster representatives. The curves represent the frequencies with which a given RMSD value is found among all distances calculated between conformations coming from the trajectories denoted by their solvent in the legend.

To further confirm the assumption that conformations coming from trajectories with different solvents are more dissimilar to each other than conformations coming from the same trajectory, the RMSD distances between all cluster representatives are calculated. More specifically, the 19 representative protein conformations obtained previously are taken, and RMSD values between all possible pairs formed from them are calculated, considering only their α carbons. By looking at the probability distribution of these RMSD values for conformation pairs obtained from the same or from different MD trajectories, one can compare the intra- and intertrajectory similarities of protein conformations. Figure 3 shows these data grouped by the solvent pairs from which the protein conformations are obtained. For example, the solid cyan curve represents the distribution of RMSD values between protein conformations coming from the same water simulation, while the dashed yellow line represents the distribution of RMSD values between conformations from the water simulation and conformations from the benzene/water simulation. The most noticeable feature of this graph is that the intratrajectory distances are considerably smaller than the intertrajectory ones, with the solid curves being to the left of the dashed ones. There is only a single outlier benzene conformation, which is quite dissimilar to all other cluster representatives obtained from this trajectory. Therefore, the data represented in Figure 3 validate our assumption that by utilizing different solvents, a more diverse set of protein conformations can be obtained than in the case when only a single solvent (water) is employed.

### Performance of the Docking Protocol

Before performing ensemble docking calculations with the obtained set of protein conformations, first, the accuracy of the employed docking protocol is evaluated. To this end, a number of confirmed binders for the RdRp of SARS-CoV-2 are obtained from ref (14), along with the most important interacting residues for each binder. To obtain these results, the authors utilized the experimentally determined apo protein structure with PDB code 6M71. To compare the performance of AutoDock Vina, we have performed docking calculations considering only the same crystallized structure. However, there are still significant differences in the preparation of the protein structure and the execution of the docking calculations between the cited work and the one presented here. Most notably, no minimization of the structure is performed here, and all residues of the protein are considered rigid during the docking calculations, contrary to the work of Ahmad et al., where key residues around the active site were treated as flexible. Therefore, although perfect agreement with the previous simulations will not be achieved, it is expected that the published binders and their poses can be at least partially reproduced using the docking procedure adopted in this work.

The results obtained with AutoDock Vina and those from ref (14) are compared in Table 1 for the seven ligands listed in the first column. This subset of the binders presented in the original article comprises the ligands for which a 3D structure could be obtained from the ZINC database. In addition, the second column of the table shows the most important interacting residues reported for each of the binders. The results returned by the AutoDock Vina simulations performed by us are given in the last two columns of the table. Specifically, the rank of the best poses returned by Vina, where the binder interacts with the residues given in the second column, is reported. Moreover, in the fourth column, the total number of found poses—out of the 20 most important ones—which interact with the specified residues is given. It can be seen, that there are only two ligands (benzquercin and pegamotecan), out of the seven considered, for which AutoDock Vina does not find the pose reported in the literature. For these two ligands, a region near the active site of the protein is clearly favored by Vina. For the other five ligands, the pose predicted in the literature is found by Vina as well, and in three cases it is among the top five returned poses. Moreover, in the case of three ligands, the predicted interacting residues are reproduced by more than one pose, indicating the stability of these results. Since the main focus of this study is not on the accuracy of the docking calculations in itself, but rather on the investigation of the effects of cosolvents and protein dynamics on the docking results, the proposed docking procedure is deemed suitable and is, therefore, utilized throughout the rest of our work.

**Table 1:** Comparison of the Results of AutoDock Vina to the Binding Poses Reported in the Literature^a^

ligand	reported interacting residues	rank of first matching pose	number of matching poses
ornipressin	ASP760, THR591, ASP865, GLN815, SER814, CYS813, GLU811, TYR619	1	8 (40%)
benzquercin	ARG553, LYS798		0 (0%)
cisatracurium	ARG553, ARG555, GLU811	2	1 (5%)
ditercalinium	ASP623, ASP760	9	1 (5%)
examorelin	ASN691, HIS810	15	3 (15%)
nacartocin	LYS798	4	3 (15%)
pegamotecan	LYS621		0 (0%)

a The data in the first two columns of the table are reproduced from Tables 1 and 2 of ref (14), originally published by Ahmad et al. The third and fourth columns show the rank of the best pose and the total number of poses returned by AutoDock Vina that interact with the residues shown in the second column.

### Ensemble Docking Simulations

#### Binding Free Energy Criterion

**Figure 4:**
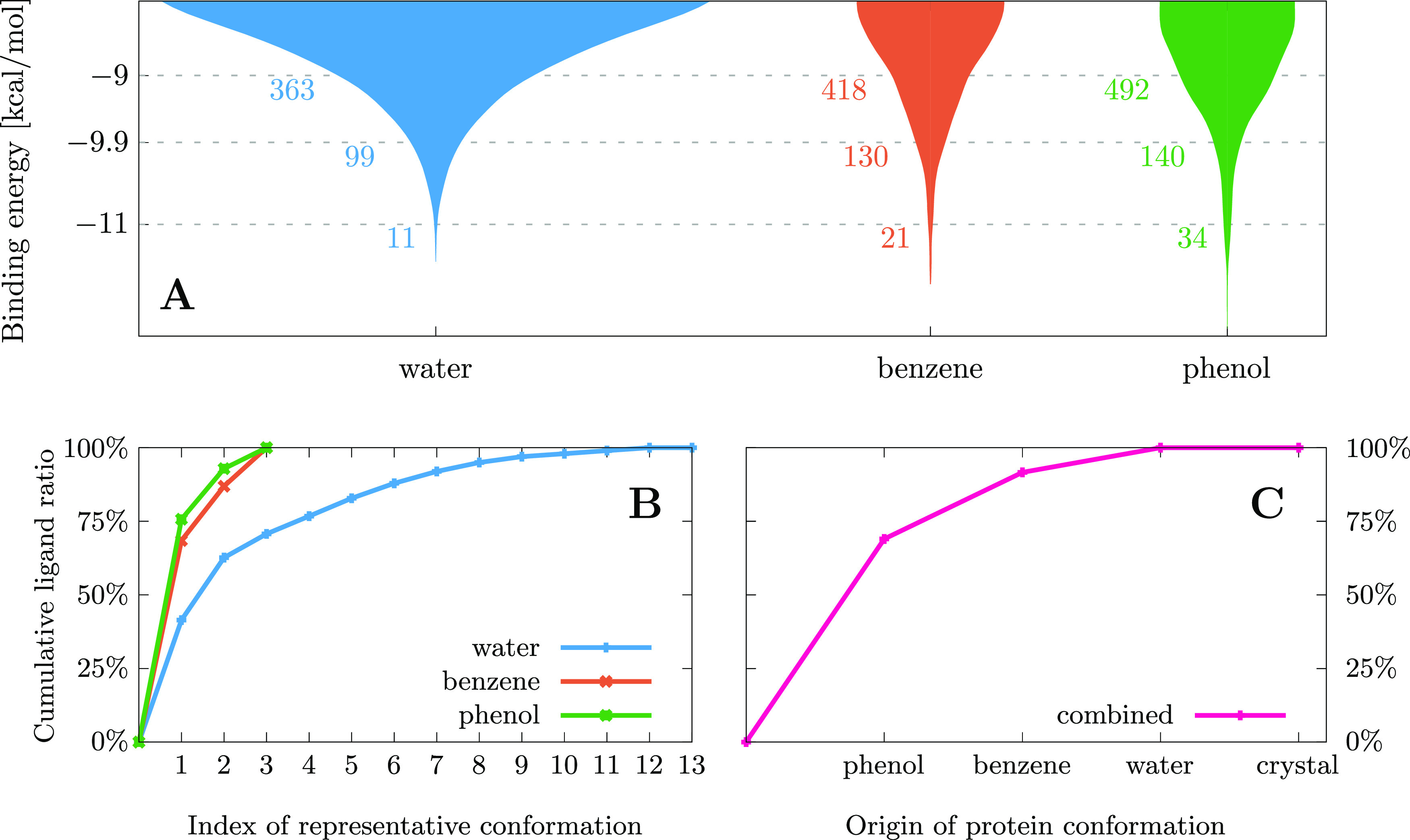
(A) Distribution of the ligand binding energies as calculated by AutoDock Vina. The numbers to the left of the distributions indicate the number of unique ligands that have a binding energy lower than the given value in the vertical axis for at least one of the conformations obtained from that solvent. Note that the distribution widths cannot be directly compared. (B) Cumulative ratio of the ligands found if only a subset of the protein conformations from each trajectory is considered for docking. (C) Cumulative ratio of the ligands discovered by considering each solvent trajectory or the apo crystal structure. The cluster representatives or trajectories on the horizontal axes are ordered in decreasing importance, from most to least new ligands discovered.

After the adeptness of the docking protocol has been verified, the distribution of the binding free energies obtained from docking the 1957 investigated drugs to the 19 representative protein conformations is examined. The distributions of the best binding energies of each protein conformation–ligand pair are shown in Figure 4A, separately for the three types of MD trajectories. The most noticeable feature of this plot is that the distributions of the two cosolvent trajectories extend to lower binding energies (or larger binding energies in absolute value), than that of the simple water trajectory. Accordingly, the number of unique ligands with favorable binding energies are larger for the cosolvent trajectories as can be observed from the colored numbers immediately to the left of each distribution on the figure. For example, considering a binding energy threshold of −9.9 kcal/mol provides 99 binders with energies larger (in absolute value) than the threshold for the water simulations and a larger number for the cosolvent simulations: 130 for benzene and 140 for phenol. Therefore, the use of nonpolar solvents leads to the binding of ligands that are better ranked than the ligands from the standard simulations in pure water. As will be discussed later, this is explained, at least in part, by the opening of transient pockets that are only accessible when the nonpolar probes interact with them. The presence of such a general trend likely indicates some significant differences in the protein conformations utilized during the docking calculations. For example, it might happen that one or more binding regions, especially those containing hydrophobic residues, are more accessible to ligands in the conformations obtained from the cosolvent trajectories than in those obtained from the water solvated trajectory, and therefore, the binding energies are more favorable in the cosolvents. The larger druggability of some pockets when the nonpolar cosolvents are used in the simulations will be discussed in more detail later.

Since the binding energy of a given ligand is very strongly dependent on the conformation of the target protein, it is expected that some ligands bind strongly only to specific protein conformations, which are not present in all of the solvent situations. In fact, it has been demonstrated in Figure 3 that the different solvent trajectories sample markedly dissimilar protein conformations. Consequently, it can be expected that some strong binders can only be found when the docking is performed with a specific solvent. When this is the case, then, one should observe that docking to one or even to just a few conformations is not sufficient to find all of the best binding ligands for a given protein. This would also mean that utilizing a well-constructed ensemble of protein conformations during the docking calculations is beneficial because it aids the discovery of new binders and better binding poses. To test this hypothesis, the selection criterion for choosing the best binding ligands is set to −9.9 kcal/mol binding energy, as this arbitrary threshold selects sufficiently many ligands for each trajectory—99, 130, and 140 for water, benzene/water, and phenol/water, respectively—from the 1957 initially docked in the protein, as can be seen in Figure 4A.

The binders selected in this way are subsequently grouped according to which cluster representative protein structures they bind to. Note that a ligand can be in multiple groups simultaneously if its binding energy is lower than −9.9 kcal/mol for more than one protein conformation. Next, the cluster representatives are ordered in descending order according to the number of ligands they bind. Finally, the representative protein conformation with the highest number of binding ligands is selected, and its ligands are removed from the lists of all other conformations, which happen to also bind that ligand. This last step is repeated until all conformations and all binding ligands are accounted for. From the data obtained in this manner, cumulative ligand binding plots are created and displayed in Figure 4B,C. These diagrams visualize the number of protein conformations which are necessary to find a certain percentage of all of the best binding ligands, discovered through any of the cluster representatives. In Figure 4B, the ligands discovered by different solvent trajectories are separated, and the cluster representatives are ordered on the horizontal axis in decreasing importance (from many to a few new ligands discovered). It can be observed that with a single protein conformation, only about 75% of all binders of the cosolvent trajectories and about 40% of the binders of the water solvent trajectory would have been discovered, even if the conformation with the most binders would have been utilized. Moreover, all protein conformations have unique ligands that only bind to them, and not to other conformations of the given trajectory. These results, therefore, validate the increased computational costs of docking to an ensemble of protein structures, as they show that significantly more binders can be discovered by utilizing multiple protein conformations than in the case of a single considered structure.

Figure 4C shows a similar curve but, instead of considering the ligands of the different protein conformations separately, they are merged together for each solvent. In this case, the horizontal axis displays the various solvents that the conformations were obtained from. From this plot, it can be observed that every MD trajectory for a specific solvent is able to find unique ligands that do not bind to conformations of the other trajectories, further validating the use of multiple solvents. The last data point of this plot corresponds to the binders discovered by docking to the apo crystal structure of the protein. It is clear that no new ligand is found by docking to this structure, that would not have been already found by one of the conformations obtained from the MD trajectories. This observation indicates that the trajectories sample a wide range of conformations, and can even account for those ligands that would bind to the crystallized protein structure. In addition, Figure 4C also shows that, despite using a very large search space (the entire protein) in the docking simulations, the results are well converged because the cosolvent benzene and phenol simulations already account for most of the docked ligands, even when the docking water simulations take into account 13 different protein conformations.

#### Binding Free Energy Gap Criterion

A common approach to selecting the most promising binders from the candidate ligands in a VS campaign is to consider the binding free energy estimate calculated by the docking program, as was done in the previous section. However, the inaccuracies of the binding energy estimates calculated by such programs are well known.^89^ Additionally, it is noted that this binding energy is naturally dependent on the size of the ligand being considered. Since ligands with a high number of atoms can benefit from more interactions between their atoms and those of the protein, this metric makes the selection of ligands that contain only a small number of atoms very unfavorable interacting moieties. These imperfections of the binding energy estimate make it a somewhat unreliable criterion for the selection of the best binding ligands. To devise an improved metric of the ligand interaction strength, one commonly used idea is to correct for the entropic terms missing from most scoring functions, by accounting for the spatial distribution of the docked poses.^90,91^ However, in the case of AutoDock Vina, the obtained best poses are first clustered by the program, and only the cluster representative poses are returned,^56^ which makes the above-mentioned pose distribution-based corrections unfeasible. In this section, an alternative approach to enhance the selection of the best binders is devised and evaluated, that can be carried out relying only on the information provided by AutoDock Vina.

Our first attempts toward devising such a ligand selection criterion revolved around scaling the binding energy estimate by the number of heavy atoms in the considered ligand. Unfortunately, this approach turned out to favor small ligands too much, while the inclusion of some parameters to scale this bias down seemed too arbitrary. Consequently, the procedure presented here is based solely on the binding energies themselves. More specifically, it evaluates the binding free energy gap between the two best poses found for the ligand. The idea behind this approach is that a truly good docked pose should be something out of the ordinary compared to the myriad of other suboptimal poses. Given the scarcity of such extraordinary minima of the scoring function, it is expected that only a small fraction of the stochastic optimization runs, performed during a docking calculation, will find the corresponding ligand poses. In practice, this phenomenon could manifest itself in the form of a single docked pose with a highly desirable binding energy estimate, found among the many other mediocre poses in the results of a docking calculation. A large gap between the binding energies of the two best docked poses for a given ligand could indicate such a situation and consequently signal a true binder of the target protein. Therefore, it is proposed to categorize ligands as binders (nonbinders) based on whether the gap between their two best binding energies is larger (smaller) than a given threshold. Finally, it is possible that this criterion functions best in combination with a simple binding energy threshold, where it would serve to eliminate some large ligands that are only categorized as binders due to the bias of the latter criterion toward larger ligands.

**Figure 5:**
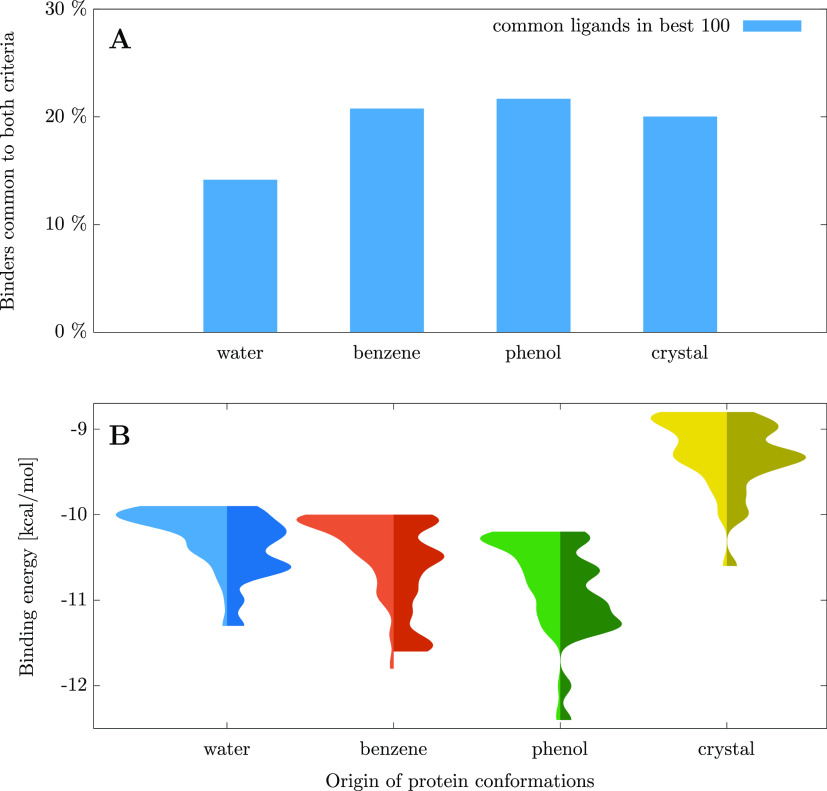
(A) Ratio of ligands selected both based on their binding free energy and the binding free energy gap between their two best poses. Ligands binding to conformations coming from different solvent trajectories (or to the crystal structure) are treated separately. For the exact definition of the ratio plotted here, see eq 1. (B) Comparison of the binding energy distributions for the ligands selected based solely on their binding energy (left half of each violin plot) and for the ligands selected both based on their binding energy and energy gap between their two best poses (right half of each violin plot). The distributions are plotted separately for the ligands of the three solvent trajectories and the crystal structure.

As the first step of evaluating the performance of this new ligand selection criterion, its preferred binders are compared to those selected based on their binding energy. If very few or no common ligands are found between the two criteria, it could indicate that this alternative selection method does not perform as expected because even though the binding energy estimate provided by the docking program is an imperfect one, in general, it will select reasonable ligands. To carry out this comparison, two sets of selection thresholds were determined for the binding energy and energy gap criteria each. The thresholds in these sets were constructed separately for the protein conformations coming from different solvent trajectories such that they select approximately 100 ligands for each trajectory. This results in thresholds between −8.8 and −10.2 kcal/mol for the binding free energy and between 0.6 and 1.2 kcal/mol for the binding free energy gap. With the best binders selected separately by the two different criteria, the ratio of common ligands can now be defined to be
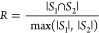
1where *S*_1_ and *S*_2_ are the sets of ligands selected by one of the criteria. In Figure 5A, this ratio of ligands selected by both criteria is plotted separately for the protein conformations obtained from each MD trajectory and the crystal structure. One can observe that about 15–20% of the best 100 ligands are common to both criteria. Even though the two criteria select ligands in entirely different manners, at least part of the ligands they favor are common to both of them. This gives us some confidence that the energy-gap-based selection criterion can indeed be suitable to augment and complement simple binding-energy-based selection methods.

After observing that a substantial ratio of the selected ligands are common for the two selection criteria, the question naturally arises: are these common ligands especially suitable binders? This would validate the use of the composite ligand selection method involving the binding energy and binding energy gap criteria. Therefore, it would be beneficial to confirm that the ligands selected based on the composite selection criterion exhibit a favorable binding energy as well, thus further increasing the confidence in the adeptness of this ligand selection method. To investigate this, the normalized distribution of the binding energies is plotted for the ligands present in the top 100 binders using the binding energy criterion and for the ligands which satisfy both the binding energy and energy gap criteria. The first distribution is represented by the lightly colored left half of each violin plot of Figure 5, while the second distribution is represented by the violin plots with a darker color. The figure clearly shows that the binding energies of those ligands which are common to both selections are lower (more favorable) than those of ligands selected solely by their binding energy. It is therefore confirmed that ligands satisfying both criteria tend to bind more favorably than ligands that satisfy only the binding energy criterion.

## Binding Sites of the Target Protein

In this section, the binding sites of the target protein are identified and characterized through the ligands interacting with them. To this end, a number of promising ligands are selected with the previously discussed two selection criteria. In both cases, the selection threshold is determined in a way to yield approximately 100 ligands for the water solvent MD trajectory. Then, the same threshold is employed for the cosolvent simulations. For this purpose, a binding energy threshold of −9.9 kcal/mol, and an energy gap threshold of 1.2 kcal/mol were found to be the most optimal.

**Figure 6:**
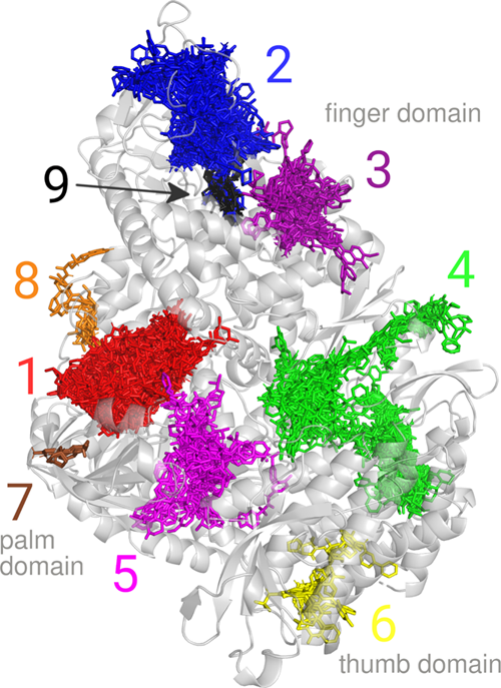
Binding sites on the protein, as identified by the visual inspection of the poses of the best binding ligands. The protein structure is the first cluster representative structure obtained from the water solvent MD trajectory. The colored molecule clusters are the superimposed binders for all cluster representative protein structures as selected by either one of the binding energy criteria. Note that only those ligands are shown which bind to one of the identified pockets, and an additional 2% of the binders which bind to other regions of the protein are hidden.

With the binders selected in this way, the most important interacting sites of the protein can be identified by visual inspection of the most favorable docked poses of the best binders. These poses are visualized in Figure 6, considering the ligands of both selection criteria together. After inspecting the docked ligands of the nine most important binding sites, interacting protein residues have also been assigned to them, collected in Table 2. It is noted that binding site number 4 identified here corresponds well to the active site of the protein, as reported in ref (14). Therefore, the interacting residues shown for this pocket in Table 2 are taken from that work.

**Table 2:** Interacting Residues of the Binding Pockets Discovered through Ensemble Docking^a^

pocket nr.	interacting residues
1	chain A: LEU172, TYR265, THR319, PRO323, THR394, PHE396, LEU460
2	chain A: ASP36, TYR38, ILE66, SER68, ASP208
3	chain A: LEU49, ASP711, ASP714, GLN773
4	chain A: ASP618, CYS622, ASN691, ASN695, MET755, ILE757, LEU758, SER759, ASP760, ASP761, ALA762, VAL763, GLU811, PHE812, CYS813, SER814
5	chain A: ASN447, chain B: PRO133, ASP134, TRP182, PRO183, chain B: LYS27
6	chain A: ASN414, ASN416, ASP418, VAL844, chain C: ILE68, chain D: ARG111
7	chain B: TYR138, THR145, TRP154, GLU155, LEU169
8	chain A: GLU254, ASP269, LYS272, ARG285
9	chain A: GLN292, THR293, LEU302, ASP303, ARG305, LEU470

a The interacting residues of the active site, which corresponds to the fourth pocket in the numbering of this work, are taken from Section 1.1 of ref (14) by Ahmad et al. The chain identifiers and residue numbers are consistent with the numbering of the structure with PDB identifier 6M71.^73^

After the binding sites of the protein have been identified, the number of ligands binding to each of them are counted and presented in Table 3, separately for the MD trajectories with different solvents, and for the apo crystal structure, and considering the binding energy and binding energy gap criteria as selection methods. Overall, the nine identified pockets are responsible for 97–99% of the best binders found for every trajectory, which clearly highlights the importance of these regions of the protein surface for potential subsequent targeted VS campaigns. It is most interesting that some of the identified pockets only bind ligands if specific cosolvents or even a specific ligand selection method is employed. This observation shows how the presence of cosolvent probes significantly influences the protein structure so much so that it can lead to the opening or the closing of certain pockets. Regarding the population of the active site (pocket 4), on the one hand, it is reassuring to see that multiple binders are found, with all three solvents considered, as this site has already been identified as druggable.^14−17^ On the other hand, no binders are found for this site if the crystallized, apo protein structure is employed in the docking calculations. This fact emphasizes the importance of protein flexibility during ligand uptake and makes it clear why docking to a single, crystallized, apo protein structure can be inadequate to utilize the full power of docking calculations and VS.

**Table 3:** Distribution of the Binders, as Selected by the Two Ligand Selection Criteria, across the Various Binding Sites of the Protein^a^

	binding energy criterion				energy gap criterion			
pocket	water	benzene	phenol	crystallized	water	benzene	phenol	crystallized
1	56	8	99	2	31	3	9	1
2	9	67	22	1	25	13	11	0
3	0	11	4	3	0	3	2	1
4	4	29	9	0	3	10	2	0
5	29	2	5	0	19	0	0	0
6	0	6	0	0	1	7	0	1
7	0	4	0	0	0	5	0	0
8	0	0	0	0	4	2	0	0
9	0	0	0	0	8	0	0	0
other	1	3	1	0	5	0	0	0
total	99	130	140	6	96	43	24	3

a Ligand counts are reported separately for each cosolvent utilized to obtain the protein conformation to which the ligand binds and the apo crystal structure. For the definition of the pocket numbering, see Figure 6 and Table 2.

Considering now the other binding sites of the protein, the most highly populated pocket is either the first or the second one, depending on the cosolvent and binder selection criterion employed. Among these, the first identified pocket is especially promising, as ligands bound here tend to be very well buried in the protein (see Figure 6). The fact that these two pockets are populated by a relatively large number of ligands, regardless of the solvent utilized to obtain the protein conformations, signals that they are quite stable and are open in most of the conformations regularly visited by the protein. On the other hand, the sixth and seventh pockets are almost exclusively populated with a few ligands when protein structures obtained from the benzene cosolvent MD trajectories are considered. It seems logical that the cause of this phenomenon is that the benzene probes were able to induce some conformational local changes in these regions of the protein. These latter two pockets are therefore more likely to be cryptic pockets, open only in rarely visited protein conformations or in the presence of binders.

For the third pocket, similar results are found as for the sixth and seventh ones, with the exception that some ligands are also selected for the conformations obtained from the phenol cosolvent MD trajectory. The fact that on top of the benzene cosolvent molecules, the more polar phenol probes were also able to open this pocket to a certain degree, could be connected to the higher ratio of hydrophilic residues around this binding site (see Table 4). Finally, the fifth, eighth, and ninth pockets all seem to be more open in the conformations coming from the water solvent trajectory, with the latter two only having ligands if the energy gap ligand selection criterion is considered. As can be seen in Table 4, the residues around these pockets also tend to be more hydrophilic than in the case of some other sites. Remarkably, these pockets are also not present in the crystallized apo structure. It is therefore very likely that some reorganization of the neighboring residues accounted for during the conventional MD calculations with water as the solvent, are necessary for their opening.

**Table 4:** Ratio of Hydrophobic Residues Near Each Pocket, along with the Cosolvent with Which the Protein Conformation Which Produced the Most Binders for That Pocket Was Obtained^a^

pocket	total no. of residues	hydrophobic residues (%)	cosolvent
1	34	47	phenol
2	35	34	benzene
3	14	21	benzene
4	19	58	benzene
5	26	31	water
6	9	55	benzene
7	6	50	benzene
8	4	0	water
9	9	33	water

a The assignment of residues to each pocket is discussed in the main text.

Next, it is interesting to investigate the reasons behind the different protein behavior in the three solvent scenarios. In particular, the presence of different intermolecular interactions between the protein and the solvent, such as stacking interactions and hydrogen bonding, likely influence the protein dynamics. These interactions are closely related with the polarity of the cosolvents and presumably with the polarity of the pockets. Therefore, it is assessed whether or not the polarity of the cosolvent molecules correlates with the hydrophilicity of the binding pockets that are open during the MD trajectory calculated with that cosolvent. To perform this analysis, the residues nearest to the nine binding sites identified in the previous section are selected. This is done by first selecting the protein conformation for which the highest number of binders are found for the pocket in question. Then, those residues which have atoms not farther than 3 Å from one of the atoms of one of the binders are chosen. Then, the selected protein residues are categorized as either hydrophilic or hydrophobic based on the results presented in ref (92). The ratio of hydrophobic residues for each pocket, along with the cosolvent with which the most populated protein conformation is obtained for that pocket, is shown in Table 4. It is noted that other methods for selecting the nearest residues for each binding site have been experimented with (*e.g*., defining a center for each pocket and defining a distance threshold from that point), and these methods yielded very similar results to those presented here.

Looking at the hydrophobicity data in Table 4, it is apparent that the pockets which were most open in the protein conformations coming from the water solvent trajectory all have low hydrophobic residue ratios. In particular pockets 8 and 9, which were previously seen to bind ligands almost exclusively if protein conformations from the water solvated trajectory are considered (see Table 3), have a low ratio of hydrophobic residues. On the contrary, the most apolar cosolvent, benzene, opens the most hydrophobic pockets best. More specifically, pockets 6 and 7, which were only discovered with the benzene cosolvent protein conformations, both have a hydrophobic residue ratio of at least 50%. Pockets 2 and 3 are also most populated with the benzene cosolvent protein conformations, even though both of them are decidedly hydrophilic in character. However, it is worth noting that these pockets are also well populated when protein structures from trajectories with water and especially with phenol are considered. Based on the above results, a link between the polarity of the solvent, and the hydrophilicity of the pockets opened by said solvent can be confidently established. This highlights once again the usefulness of employing cosolvent MD simulations for the generation of protein conformations in ensemble docking: by utilizing apolar cosolvent probes, new, more hydrophobic binding sites can be opened that would otherwise not be discovered.

**Figure 7:**
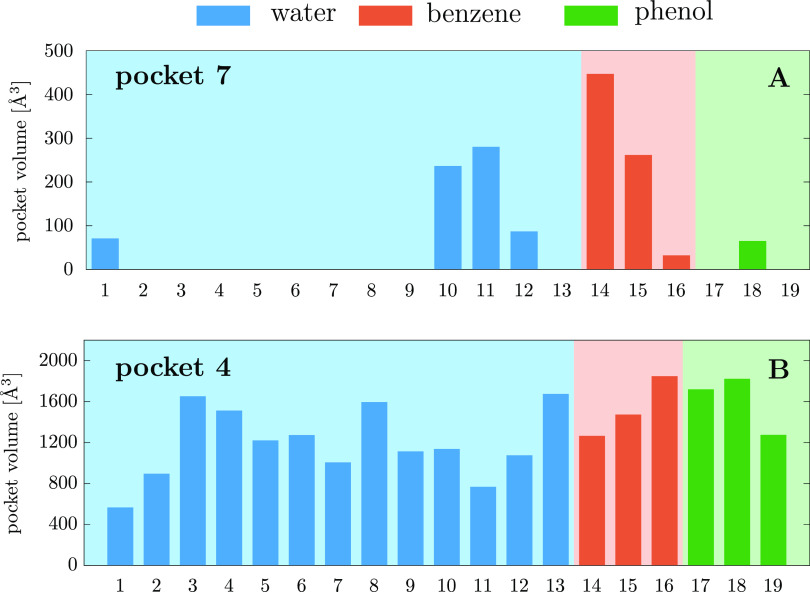
Volumes of pockets 4 (A) and 7 (B) calculated for all considered protein conformations. Pocket volumes were calculated with the mdpocket program. For the definition of these binding pockets, see Table 2 and Figure 6.

Finally, the volumes of a stable and a transient pocket across conformations of the ensemble are compared to identify further characteristics of the different pocket types. The pocket volumes are calculated by mdpocket and are shown in Figure 7 for all 19 protein conformations. Looking at the top plot of this figure, where the volumes of the transient pocket 7 are shown, one can observe that the structures obtained from the benzene MD trajectory harbor pocket conformations with the largest volumes, in one case reaching more than 400 Å^3^. This observation is to some extent in line with the fact that ligands are only able to bind to this pocket when the protein conformations obtained from the benzene trajectory are considered (see Table 3). In general, it is clear that this pocket has a nonzero volume only in a small subset of the complete conformational ensemble, further confirming its transient nature. Furthermore, the ability of the benzene cosolvent simulations to provide open pocket conformations is again demonstrated. It must be noted that mdpocket calculates nonzero volumes for some protein conformations obtained from the water solvent trajectory as well, indicating the presence of an open pocket in those conformations. However, the docking calculations found no ligands for the water solvent trajectory, not even for these conformations. This indicates that simple descriptors, such as the volume, although valuable, are not perfectly reliable and cannot be considered a substitute to the more accurate explicit docking calculations. In other words, the ability of a pocket to accommodate binders cannot be quantified by just looking at simple structural descriptors.

In comparison to the results found for pocket 7, one can observe a more homogeneous pocket volume distribution across the various protein structures for the stable pocket 4. However, the fluctuation between protein conformations is still large with the volume of the active site pocket ranging between 600 and 1700 Å^3^. Comparing the two binding sites further, pocket 4 is found to have a much larger volume than the transient pocket 7 in all frames. The main similarity between these pockets is that their largest respective volumes are calculated when conformations coming from the benzene cosolvent trajectories are considered. However, this is not surprising because both pockets present the largest number of binders for the benzene/water simulation, as was shown in Table 3. That being said, the difference in pocket volume between the water solvent and benzene cosolvent or between the phenol and benzene cosolvents trajectory frames is far less significant in the case of the active site. This agrees with the fact that several binders are found to bind pocket 4 for the water and phenol/water trajectories, while this is not the case for pocket 7, which is populated only when benzene is used as cosolvent. The ability of cosolvent MD trajectories to provide higher pocket volumes in comparison to traditional MD simulations for both stable and transient pockets is worth mentioning.

## Conclusions

In drug discovery projects, the technique of VS has proved to be especially useful, contributing to the discovery of hundreds of small-molecule drug candidates by performing docking calculations to a given protein structure with thousands of ligands. Owing to its numerous successes, considerable efforts are expended to achieve further improvements in its accuracy and usability. For example, the introduction of conformational sampling has been shown to be crucial to improve the efficacy of VS campaigns. In the present work, we have considered conformational sampling by performing ensemble docking calculations on different protein geometries generated by classical MD simulations. To further improve the sampling of the configurational space, the simulations were evolved in three different solvent scenarios: on top of the usual water solvent, benzene/water and phenol/water mixtures were utilized. Due to the COVID-19 pandemic that has already claimed millions of lives around the world, the simulations were performed for the RdRp protein of the SARS-CoV-2 virus as a target of almost 2000 FDA-approved drug molecules, in the hopes of revealing information, especially from the methodological point of view, that can contribute to the development of effective treatments.

The ensemble of protein geometries employed in the docking calculations was generated by density-based clustering of the MD trajectories evolved in the three solvents. The clustering analysis provided 13, 3, and 3 representative conformations for the simulations in water, benzene/water, and phenol/water, respectively. These cluster representative frames selected from different cosolvent trajectories proved to harbor protein conformations with meaningful differences, as evidenced by their large RMSD distances from each other. This result in itself highlights the importance of running MD simulations with different cosolvents since the different protein conformations obtained could accommodate different binders compared to the traditional dynamics simulations run in pure water as the solvent.

Ensemble docking calculations were performed utilizing the 19 representative protein conformations and the crystal structure, together with a set of approximately 2000 FDA-approved drug molecules. The best binders were identified attending to two different selection methods based on the binding free energy and the binding free energy gap between the two best docked poses of a particular drug. The second selection method is intended to help in finding the ligands with the best interacting chemical moieties, regardless of the size of the ligand since the simple binding energy criterion might overestimate the importance of large ligands. It was found that both approaches select a reasonable ratio of common ligands. Most importantly, those ligands selected by both methods presented the most favorable binding energies of all of the considered ligands. Therefore, the importance of using different binder selection methods—and a combination of them—to propose promising protein binder candidates was demonstrated.

Subsequently, utilizing either of the two selection criteria, the best binders of the protein were selected. By inspecting the poses of these ligands, nine important binding sites (including the already known active site) of the protein were identified that harbored the best pose for over 98% of the best binders. The pocket search was dependent on the solvent and on the binder selection criteria, indicating once again that the combination of different approaches can provide better results in VS campaigns. In addition, the importance of conformational sampling was evidenced since only four pockets were identified by running the docking simulations employing the crystal apo structure of the protein, instead of the 19 representative conformations. In addition, it has been shown that the new pockets identified when cosolvent simulations are evolved lead to the discovery of binders that present a larger binding energy than the ones obtained in pure water.

By analyzing the populations of the binding pockets across different MD trajectories they were separated into two groups, corresponding to stable and transient binding sites. It was found that stable pockets are open in many or all frames of the various MD trajectories, and they bind a relatively large number of ligands. On the contrary, transient or cryptic binding sites were found to be open only in a limited set of protein conformations, often originating from the same cosolvent MD trajectory calculated with an apolar cosolvent. In fact, a correlation between the polarities of the pockets and solvents was found.

To understand the effects of cosolvents better, the protein conformations around two selected binding sites were compared between representative structures originating from different MD trajectories. One of these selected pockets was the active site of the protein, which was characterized as a stable pocket, and the other one was a transient pocket distal to the active site. Considering first the transient pocket, it was found that only the apolar cosolvent benzene was able to induce conformational changes which lead to an increase of the pocket volume, and therefore to the opening of the binding site. Looking at the active site, it was revealed that this region of the protein is more conserved since a more homogeneous pocket volume distribution across the protein structures and the solvents was observed. However, despite this relatively constant volume behavior for the active site, it was found that the effects of cosolvents are significant on both cryptic and well-known stable pockets, with more druggable protein conformations visited during cosolvent MD simulations than either those found in the crystallized protein structure or those visited during traditional MD trajectories with water as the solvent.

In summary, the use of dynamics simulations that introduce nuclear motion, the introduction of different solvent combinations that further improve sampling, and the application of different criteria to select the best binders from docking calculations provided a larger variety of potential protein binding sites and drug candidates for the SARS-CoV-2 RdRp protein than static docking calculations. The discovery of these additional binding pockets different from the active site and strong binders could be irrelevant for the biological function of the protein and, therefore, for the discovery of new antiviral agents, but could also be important by decreasing the affinity for ligands at the active site leading to allosteric inhibition. This can be elucidated by running additional simulations aimed to investigate the effect of ligand binding to different pockets on the topology of the active site of the protein. Thus, an efficient full theoretical drug design procedure would involve the evolution of cosolvent dynamics and ensemble docking calculations, followed by the selection of the best binder/pocket complexes for which the possibility of protein inhibition would be explored by further dynamic simulations. Such a protocol could be applied to future VS projects aimed to search for drug candidates to fight against COVID-19 and other diseases.

## Supporting Information Available

Details about the choice of the clustering parameters and procedure (PDF)

## Notes

The setup of the systems was carried out with the help of the AmberTools19 scripts, which are available in the Amber 18 program package^75^ (https://ambermd.org/), and with packmol(80) (http://leandro.iqm.unicamp.br/m3g/packmol/home.shtml). The classical MD trajectories were evolved with the CUDA version of the pmemd program of Amber 18.^75,83^ Then, the clustering of the trajectories was performed with the cpptraj program^84^ (https://amber-md.github.io/cpptraj/CPPTRAJ.xhtml) of Amber 18. The mol2 files of the drug structures downloaded from the ZINC database^85^ were converted to pdb format by the openbabel program^86^ (http://openbabel.org/wiki/Main_Page). Then, the protein and ligand structures were prepared for docking calculations by scripts included in the AutoDockTools 4 distribution^87^ (http://autodock.scripps.edu/resources/adt), and the docking calculations were run by Autodock Vina 1.1.2^56^ (http://vina.scripps.edu/). The pockets found in the docking calculations were analyzed with the mdpocket program,^88^ part of the fpocket distribution (https://github.com/Discngine/fpocket). The simulations were visualized with Visual Molecular Dynamics^93^ (https://www.ks.uiuc.edu/Research/vmd/).
