# Supplementary material for: Cosolvent and Dynamic Effects in Binding Pocket Search by Docking Simulations
Source: J Chem Inf Model. 2021 Nov 3;61(11):5508–23. doi: 10.1021/acs.jcim.1c00924 (PMC8659376; doi:10.1021/acs.jcim.1c00924)
Supplement: Supplementary file 1 — ci1c00924_si_001.pdf [file ci1c00924_si_001.pdf]

Supporting Information for "Cosolvent and Dynamic Effects in Binding Pocket Search by Docking Simulations"

P. Bernát Szabó,[†,‡] Francesc Sabanés Zariquiey,[†] and Juan J. Nogueira[*,‡,¶]

†*Department of Chemistry, KU Leuven, Celestijnenlaan 200F 3001 Leuven, Belgium*

‡*Department of Chemistry, Universidad Autónoma de Madrid, Calle Francisco Tomás y Valiente, 7, 28049, Madrid, Spain*

¶*IADCHEM, Institute for Advanced Research in Chemistry, Universidad Autónoma de Madrid, Calle Francisco Tomás y Valiente, 7, 28049 Madrid, Spain*

E-mail: juan.nogueira@uam.es

Choice of Clustering Parameters

The tuning of the parameters of the `dbscan` algorithm can be performed in different ways, for example, by analyzing the evolution of the number of clusters with changing ε, or the preparation of so called k-dist curves, in which case suitable ε values can be obtained by considering the first "valley" of the curve.[1] In this work the clustering parameters were chosen by systematically varying the values for these parameters to see which combination yields the most optimal clustering by analyzing some clustering metrics. The computation of the RMSD distances between all frames of a trajectory is much more demanding if on top of the alpha carbons, all other heavy atoms are considered as well. To speed up these computations, the technique of sieving is utilised: only every other frame is considered

explicitly during the clustering, the remaining frames are simply added to the cluster with the cluster representative most similar to them. The number of frames utilised during the clustering was 11000, 11400 and 10400 for the water, phenol/water and benzene/water trajectories. To measure the quality of the clustering, four metrics are utilised. Two of these, namely the Davies–Bouldin index (DBI) and the pseudo-F statistic (pSF), aim to compare the intra- and intercluster variances, with a small intracluster and large intercluster variance indicating good quality clustering.[2] Since both of these scores are heavily influenced by the number of obtained clusters, the comparison of their absolute values between different MD trajectories has limited meaning. Instead, the trends arising in these metrics through the systematic variation of the clustering parameters can be interpreted to optimise these parameters. At this point it is useful to mention that low values of DBI and high values of pSF are desirable. The other two descriptors utilised to describe the quality of the clustering are the number of noise frames (frames not included in any cluster), and the number of clusters defined by the algorithm. The number of noise frames should clearly be kept low to avoid missing any important conformations only because it is visited very rarely and is therefore considered an outlier by the algorithm. The noise frames are automatically determined by the algorithm based on the supplied ε and k parameters and are defined as those frames which do not belong to any cluster and are not cluster centers themselves. Finally, while a high number of clusters is desirable, as it can result in a wider variety of protein conformations, the computational limitations of performing explicit docking calculations to each representative conformation with thousands of ligands should be kept in mind.

On Figure S1, the descriptors of the water solvated trajectory clustering can be seen for the case when only the alpha carbons are considered during the RMSD distance calculations. As it can be seen, considering ε values larger than 1.2 Å leads to a single obtained cluster (Figure S1D), for which the clustering descriptor metrics cannot provide a meaningful value. Since a single cluster is clearly not ideal, these large ε values do not need to be considered during the search for the optimal parameters. Focusing instead on parameter k, the most

significant differences between the different values for this parameter can be discovered on the pSF bahaviour (Figure S1B). Here, the curves with k= 4 or 6, reaching their peak at ε=1.1 Å, are clearly superior to the other two. Contrary, the variation of k has much more limited effects on DBI, as shown in Figure S1A. In fact, all DBI curves are more or less constant if ε is smaller than or equal to 1.1 Å, at which point the DBI values drop rapidly and become zero at 1.2 Å. Considering the behaviour of the pSF and DBI descriptors, values of ε=1.1 Å and k=4 are promising candidates to be the optimal choice. Further advantages of this choice can be seen by looking at Figure S1C,D: the number of noise frames stay below 2 %, while a reasonable number of clusters (thirteen) is obtained. The thirteen clusters resulting in thirteen representative protein conformations is deemed suitable number both because docking to these conformations represents a manageable computational challenge, and because similar numbers of clusters have been reported in the literature for comparable MD trajectories.[3,4]

As a comparison, similar descriptors are calculated for the clusterings when all heavy atoms are considered during the RMSD calculations. The trends observed in this case look very similar to those discussed when only the alpha carbons are considered, therefore these plots are omitted. The only notable difference is that significantly larger ε values are needed to obtain similar results as in the case when only the alpha carbons are considered. This phenomena can be explained if higher mobility is assumed for the non-backbone heavy atoms of the protein in comparison to the alpha carbons. Since no clear advantage of the all heavy atom clustering is found, the significantly increased computational costs of considering much more atoms for the RMSD calculations make this type of clustering an inferior option compared to considering only the alpha carbons.

The performance of the clustering algorithm is also examined on the benzene cosolvent trajectory, with only the alpha carbons considered for the RMSD distance calculations, to investigate any potential differences in the quality of the clustering due to the presence of cosolvent probes during the simulation. The same descriptors as in the case of the water

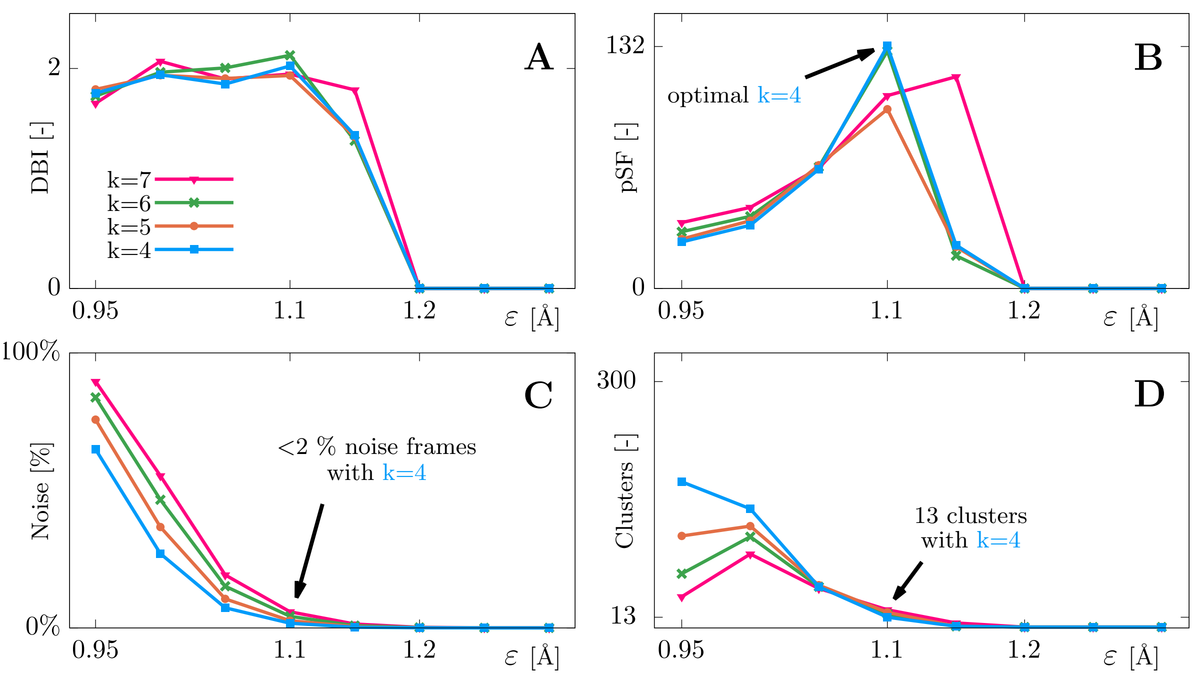

Figure S1: Clustering descriptors utilised for the tuning of the `dbscan` parameters: (A) the Davies–Bouldin index (DBI), (B) the pseudo-F statistic (psF), (C) the percentage of noise frames, and (D) the number of clusters. The descriptors were obtained by clustering the MD trajectory with water as the solvent and considering only the alpha carbon atoms for the RMSD calculations. The ε parameter in units of Ångströms are shown on the horizontal axes in all cases, while on the vertical axes the various unitless descriptors are shown.

solvated trajectory are plotted on Figure S2. Contrary to the water trajectory, in this case the number of clusters does not decrease to a single one at higher ε values, but instead is saturated at three, see Figure S2D. As a consequence, the DBI and pSF values do not vanish for these values of ε (Figure S2A,B). Instead, a sudden shift can be observed between ε=1.0 Å and 1.1 Å for both metrics, while for values higher or lower than these, the curves are more or less constant. The facts that this shift is occurring near $\varepsilon = 1.1$ Å, and that this value is already in the more favorable interval for both metric curves (small values of DBI and high values of pSF), highlight the attractiveness of choosing 1.1 Å as the value of the ε parameter. The pSF value at $\varepsilon = 1.1$ Å of the curve associated with $k = 4$ is again one of the best along with $k = 5$. Figure S2C,D reveals no surprises: the number of noise frames is negligible with the parameter values being considered, while the number of clusters stagnates around the reasonable value of 3 and increases sharply only for ε values lower than 1.0 Å. Similar plots have been created for the trajectory with phenol as the cosolvent, however it showed very similar characteristics as this one, therefore it is not displayed here. To summarise, the clustering parameters values of $\varepsilon = 1.1$ Å and k=4 prove to be ideal choices, as they result in a clustering that is suitable for our purposes for all types of trajectories considered. The resulting clustering yielded 19 total representative protein conformations (13 from the water, 3 from the benzene/water and 3 from the phenol/water trajectories), which were utilized during the subsequent ensemble docking calculations.

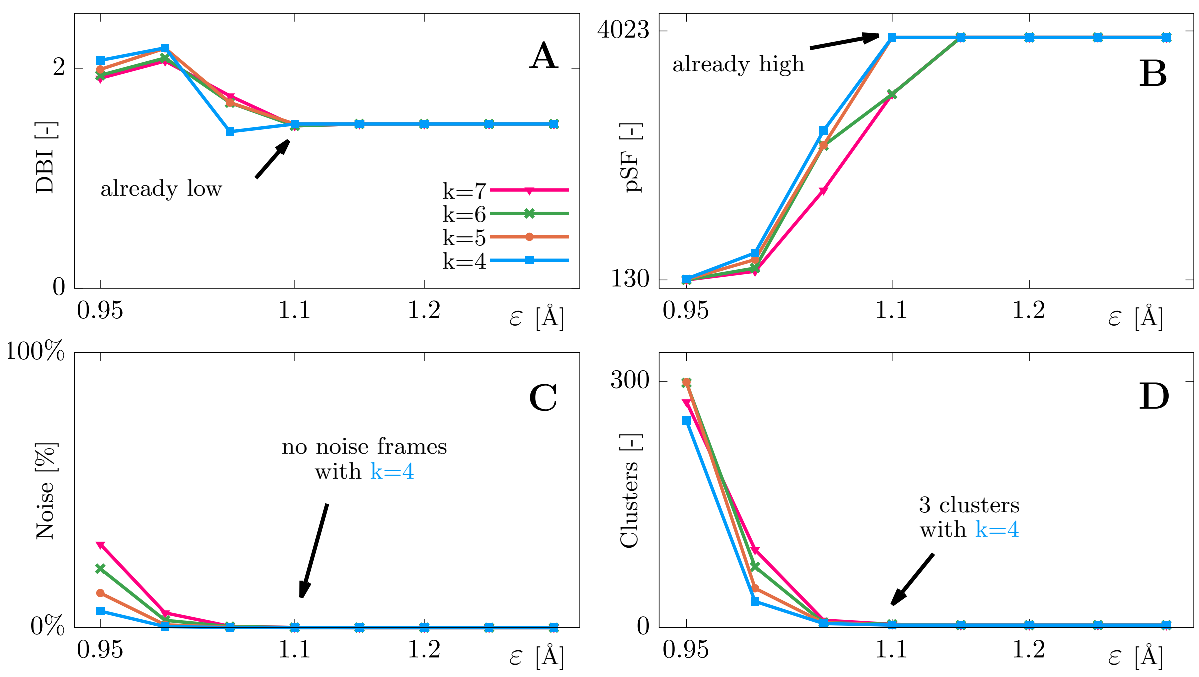

Figure S2: Clustering descriptors utilised for the tuning of the `dbscan` parameters: (A) the Davies–Bouldin index (DBI), (B) the pseudo-F statistic (psF), (C) the percentage of noise frames, and (D) the number of clusters. The descriptors were obtained by clustering the MD trajectory with benzene as the cosolvent and considering only the alpha carbon atoms for the RMSD calculations. The ε parameter in units of Ångströms are shown on the horizontal axes in all cases, while on the vertical axes the various unitless descriptors are shown.

References

(1) Kim, M.; Choi, S.-H.; Kim, J.; Choi, K.; Shin, J.-M.; Kang, S.-K.; Choi, Y.-J.; Jung, D. H. Density-Based Clustering of Small Peptide Conformations Sampled from a Molecular Dynamics Simulation. *Journal of Chemical Information and Modeling* **2009**, *49*, 2528–2536.

(2) Shao, J.; Tanner, S. W.; Thompson, N.; Cheatham, T. E. Clustering Molecular Dynamics Trajectories: 1. Characterizing the Performance of Different Clustering Algorithms. *Journal of Chemical Theory and Computation* **2007**, *3*, 2312–2334.

(3) Durrant, J. D.; Urbaniak, M. D.; Ferguson, M. A. J.; McCammon, J. A. Computer-Aided Identification of Trypanosoma brucei Uridine Diphosphate Galactose 4'-Epimerase Inhibitors: Toward the Development of Novel Therapies for African Sleeping Sickness. *Journal of Medicinal Chemistry* **2010**, *53*, 5025–5032.

(4) Durrant, J. D.; Cao, R.; Gorfe, A. A.; Zhu, W.; Li, J.; Sankovsky, A.; Oldfield, E.; McCammon, J. A. Non-Bisphosphonate Inhibitors of Isoprenoid Biosynthesis Identified via Computer-Aided Drug Design. *Chemical Biology & Drug Design* **2011**, *78*, 323–332.
